# Pan-cancer analysis of LncRNA XIST and its potential mechanisms in human cancers

**DOI:** 10.1016/j.heliyon.2022.e10786

**Published:** 2022-09-28

**Authors:** Wei Han, Chun-tao Shi, Jun Ma, Hua Chen, Qi-xiang Shao, Xiao-jiao Gao, Ying Zhou, Jing-feng Gu, Hao-nan Wang

**Affiliations:** a Department of General Surgery, Kunshan First People's Hospital Affiliated to Jiangsu University, Kunshan Jiangsu, 215300, PR China; b Department of General Surgery, Kunshan Maternity and Child Care Hospital, Kunshan Jiangsu, 215300, PR China; c Department of General Surgery, Wuxi Xishan People’s Hospital, Wuxi Jiangsu, 214000, PR China; d Department of Urology, Kunshan Hospital of Traditional Chinese Medicine, Kunshan Jiangsu, 215300, PR China; e Department of Immunology, Key Laboratory of Medical Science and Laboratory Medicine, School of Medicine, Jiangsu University, Zhenjiang Jiangsu, 212013, PR China; f Department of Pathology, Kunshan First People's Hospital Affiliated to Jiangsu University, Kunshan Jiangsu, 215300, PR China; g Oncology Department, Wuxi Fifth People's Hospital, Wuxi Jiangsu, 214000, PR China

**Keywords:** XIST, Pan-cancer analysis, Prognosis, miRNA, ceRNA, Tumor immunity

## Abstract

**Background:**

X-inactive specific transcript (XIST), it has been found, is abnormal expression in various neoplasms. This work aims to explore its potential molecular mechanisms and prognostic roles in types of malignancies.

**Methods:**

This research comprehensively investigated XIST transcription across cancers from Oncomine, TIMER 2.0 and GEPIA2. Correlations of XIST expression with prognosis, miRNAs, interacting protens, immune infiltrates, checkpoint markers, mutations of tumor-associated genes and promoter methylation were also analyzed by public databases. In addition, 98 BRCA samples were collected to investigate XIST expression and evaluate its clinicopathological value.

**Results:**

In public databases, compared to normal tissues, XIST was lower in BRCA, CESC, COAD and so on, but increased in KIRC and PRAD. Databases also showed that XIST was a good indicator of prognosis in BRCA, COAD and so on, but a bad one in KIRC, KIRP and so on. From starBase, we found 29 proteins interacting with XIST, and identified 4 miRNAs which might be sponged by XIST in cancers. Furthermore, XIST was linked with immune infiltration, especially T cell CD4+, and was related to over 20 immune checkpoint markers. Moreover, several tumor-associated gene mutations and promoter methylation were negatively related to its expression. In addition, IHC showed that XIST in BRCA was obviously lower in comparison of normal tissues and was negatively related to lymph node invasion and TNM stage.

**Conclusion:**

In summary, abnormal expression of XIST influenced prognosis, miRNAs and immune infiltration across cancers, especially BRCA.

## 1 Introduction

Neoplasm has become a global health problem and a leading cause of death worldwide, with about 20 millions new cases and 10 million deaths in 2020 [1]. Although operation, radiotherapy, chemotherapy, targeted therapy and immunotherapy are the main treatment options for carcinomas, patients at the advanced stage still have poor prognosis [2]. Therefore, exploring novel biomarkers is essential for preventing chemo-resistance and improving survival rates of cancer patients.

With a length of more than 200 nucleotides, long non-coding ribonucleic acids (LncRNAs) are a highly heterogeneous group of transcripts, and are involved in various biological functions through the epigenetic regulation of genes and the interaction with proteins and RNAs [3, 4]. Due to its regulation, abnormal expressions of LncRNAs lead to the development and progression of many diseases, especially malignant tumors [5, 6]. Therefore, more and more LncRNAs are considered as novel biomarkers to predict chemoresistance and evaluate prognosis of cancer patients [7, 8].

LncRNA X-inactive specific transcript (XIST) locates at Xq13.2 and coats the X chromosome in cis during X chromosome inactivation (XCI) [9]. Emerging investigations report that abnormal expression of XIST takes part in the regulation of various diseases, including solid tumors [10, 11, 12]. Previous researches reported that XIST could induce biological behavior and pathological appearance by interacting with several proteins and micro ribonucleic acids (miRNAs), and could play key roles in the generation, progression and prognosis of tumors [13, 14]. Recent studies have demonstrated that XIST is down-regulated in several cancers and suppresses the progression of tumors, especially breast cancer [15, 16]. However, some studies showed that XIST promoted growth and invasion of colorectal cancer cells, and silencing XIST could repress chemoresistance of acute myeloid leukemia [17, 18]. These entirely different roles of XIST in carcinomas resulted in discrepancies of its prognostic value among previous researches [19, 20].

The tumor microenvironment (TME) is a complex milieu in which immune infiltration can activate or restrain tumor progression and metastasis, which forms the tumor immune microenvironment (TIME) [21, 22]. Previous studies discovered that XIST and its downstream regulators were correlated with TIME and PD-L1 expression, and played critical roles in invasion and metastasis of cancers [16, 23, 24, 25].

Gene mutation is regarded as a crucial factor for malignant transformation and tumor progression [26]. In addition, Gene mutation, such as BRCA1 and BRCA2, affected immune cell infiltration and response to immunotherapy [26]. Abnormal expression of XIST was also related to the mutation of several tumor-associated genes [27, 28]. For example, dysregulation of XIST and 53BP1 affected the survival of breast carcinoma patients with BRCA1 mutation [27]. However, whether the mutation of BRCA1 or other genes leads to abnormal expression of XIST is still obscure.

In view of the outstanding contradictions and vagueness above, we conducted a pan-cancer analysis of XIST to elucidate its potential molecular mechanisms and prognostic roles in multiple cancers. In addition, 98 cases of breast cancer were also collected to assess the clinicopathological role of XIST in BRCA.

## 2 Materials and methods

### 2.1 Pan-cancer analysis of XIST transcription

Oncomine (https://www.oncomine.org/), Tumor Immune Estimation Resource 2.0 (TIMER 2.0, https://timer.cistrome.org/), Gene Expression Profiling Interactive Analysis 2 (GEPIA2, https://gepia2.cancer-pku.cn/) were performed to analyze XIST transcription in multiple cancers. The first database was Oncomine which consisted of more than 80,000 samples of over 20 types of cancers. The threshold in this research was set as the following criteria: gene rank: Top 10%, fold change: 2, and *P* value: 1E-4. The second one was TIMER 2.0 containing more than 10 thousand samples of over 30 cancer types from The Cancer Genome Atlas (TCGA) [29, 30]. The next one was GEPIA2 which was a newly developed database for analyzing the RNA sequencing expression data of more than 9,000 tumors and 8,000 normal tissues from the TCGA and the Genotype-Tissue Expression (GTEx) projects [31].

Names and abbreviations of types of cancers were all listed as follow: ACC: adrenocortical carcinoma; BLCA: bladder urothelial carcinoma; BRCA: breast invasive carcinoma; CESC: cervical squamous cell carcinoma and endocervical adenocarcinoma; CHOL: cholangio carcinoma; COAD: colon adenocarcinoma; DLBC: lymphoid neoplasm diffuse large B-cell lymphoma; ESCA: esophageal carcinoma; GBM: glioblastoma multiforme; HNSC: head and neck squamous cell carcinoma; KICH: kidney chromophobe; KIRC: kidney renal clear cell carcinoma; KIRP: kidney renal papillary cell carcinoma; LAML: acute myeloid leukemia; LGG: brain lower grade glioma; LIHC: liver hepatocellular carcinoma; LUAD: lung adenocarcinoma; LUSC: lung squamous cell carcinoma; MESO: mesothelioma; OV: ovarian serous cystadenocarcinoma; PAAD: pancreatic adenocarcinoma; PCPG: pheochromocytoma and paraganglioma; PRAD: prostate adenocarcinoma; READ: rectum adenocarcinoma; SARC: sarcoma; SKCM: skin cutaneous melanoma; STAD: stomach adenocarcinoma; TGCT: testicular germ cell tumors; THCA: thyroid carcinoma; THYM: thymoma; UCEC: uterine corpus endometrial carcinoma; UCS: uterine carcinosarcoma; and UVM: uveal melanoma.

### 2.2 Prognosis analysis

The correlation of XIST expression with prognosis of patients in multiple cancers was analyzed through three public databases, GEPIA2, PrognoScan (http://dna00.bio.kyutech.ac.jp/PrognoScan/index.html) and Kaplan-Meier Plotter (https://kmplot.com/analysis/). Data of PrognoScan mostly came from the Gene Expression Omnibus (GEO) database, and Kaplan-Meier Plotter utilized *Affymetrix microarray* data from TCGA [32, 33]. We used heat maps, forest plots and KaplanMeier curves to visualize the survival data of cancer patients. Overall survival (OS), disease free survival (DFS), relapse free survival (RFS), disease specific survival (DSS), distant metastasis free survival (DMFS), progression free survival and distant recurrence free survival were main outcomes. The hazard ratio (HR) and 95% confidence interval (CI) were calculated through univariate analysis.

### 2.3 Exploration of proteins and miRNAs interacting with XIST

We performed starBase (http://starbase.sysu.edu.cn/starbase2/index.php) to identify candidate proteins and miRNAs potentially interacting with XIST by Pearson correlation analyses [34]. In addition, the ceRNA network of starBase was conducted to search for possible RNAs that could compete with XIST for miRNAs binding. Next, a network of LncRNA - miRNAs - mRNAs was scheduled by the software named GEPHI.

### 2.4 Tumor immune microenvironment analysis

TIMER 2.0 was utilized to analyzed the relationship of XIST expression with the abundance of infiltrating immune cell types in multiple cancers by Spearman correlation analyses. In addition, we evaluated the correlations of XIST with immune checkpoint markers and immune cell types in each type of cancers by Spearman correlation analysis.

### 2.5 Pan-cancer analysis of representative gene mutation

We performed TIMER 2.0 to analyze the correlation of XIST expression with representative gene mutation by Spearman correlation analyses. 47 representative genes were listed as follow: AKT1, ALK, APC, AR, ARID1A, ASXL1, ATM, BAP1, BARD1, BRAF, BRCA1, BRCA2, BRIP1, CCND1, CDK4, CDKN2A, CHEK2, EGFR, EPCAM, ERBB2, ERBB3, FANCA, FAT1, FBXW7, FGFR1, KDR, KIT, KRAS, MET, MTOR, NBN, NTRK1, NTRK2, NTRK3, PALB2, PIK3CA, PTEN, RAD51C, RAD51D, RB1, RET, ROS1, SMO, STK11, TP53, TP53BP1 and TSC1.

### 2.6 Methylation analysis of XIST promoter

Firstly, EPD (eukaryotic promoter database, https://epd.epfl.ch//index.php), a set of species-specific databases of experimentally validated promoters, was performed to identified XIST promoter [35]. Then, we put the XIST promoter sequence into MethPrimer 2.0 (http://www.urogene.org/cgi-bin/methprimer/methprimer.cgi) to detect the CpG islands in XIST promoter. Another database EWAS Data Hub (https://ngdc.cncb.ac.cn/ewas/datahub/index), a data hub of DNA methylation array data and metadata, was also utilized to analyze the relationship between survival time and XIST promoter methylation level, and the relationship between XIST expression and promoter methylation level across 39 types of cancers [36].

### 2.7 Patients and breast cancer samples

Specimens from a total of 98 female invasive breast cancer patients who underwent mastectomy in Wuxi Xishan People’s Hospital from January, 2015 to January, 2020 were collected with a mean age of 55.45 ± 12.02 years. Each case consisted of breast tumor and its corresponding adjacent normal tissue, which were identified via hematoxylin-eosin (HE) staining by XJG and JFG, and were used for real-time quantitative polymerasechain reaction (RT-qPCR). This research had received the approval of Wuxi Xishan People’s Hospital Ethics Committee. Every patient signed the informed consent form.

### 2.8 RT-qPCR

Tissues were collected to isolate total RNA through Trizol regent (Thermo Fisher Scientific, USA). A total of 2 μg RNA of each sample was reverse transcribed using the HiScript III RT SuperMix for qPCR (Vazyme-innovation in enzyme technology, China). cDNA was subjected to quantitative PCR using primers specific for XIST and GAPDH. PCR primers were designed as follow: XIST, Forward primer (5’-3’), CTAAGGGCGTGTTCAGATTGT, Reverse primer (5’-3’), ACCTGCTATCATCCATCTTGC (123 bp); GAPDH, Forward primer (5’-3’), GAAGGTGAAGGTCGGAGT, Reverse primer (5’-3’), GAAGATGGTGATGGGATTTC (226 bp). RNA was quantified with a real-time PCR machine (IQ5, Bio-Rad, USA) using the ChamQ Universal SYBR qPCR Master Mix (Vazyme-innovation in enzyme technology, China). Then, we used 2^−ΔΔCt^ value to calculate the relative expression. The formula of ΔΔCt = (Ct_Tumor-XIST_ - Ct_Tumor-GAPDH_) - (Ct_Normal-XIST_ - Ct_Normal-GAPDH_).

### 2.9 Statistical analysis

Differences of levels of XIST transcription between tumors and normal samples were analyzed by *t*-tests. HRs and *P* value were calculated by univariate Cox regression model in PrognoScan, and by log rank test in GEPIA2 and Kaplan-Meier Plotter. Pearson or Spearman correlation analyses were utilized as above. The ROC curve was used to test the accuracy of XIST expression in the diagnosis of breast cancer and the maximum value of Uden's index (Uden's index = sensitivity + specificity-1) was used to identify the cut-off value. According to the cut-off value, we divided these patients into two groups, named as “positive group” and “negative group”. Then, Chi-squared test (χ^2^) was utilized to compare clinicopathological parameters in two groups by SPSS 20.0. The comparison of XIST in 98 samples between breast tumors and normal tissues was conducted by Graphpad 6.0. Above all, *P* < 0.05 was set as a statistically significant threshold.

## 3 Results

### 3.1 The RNA expression level of XIST in multiple cancers

**Figure 1:**
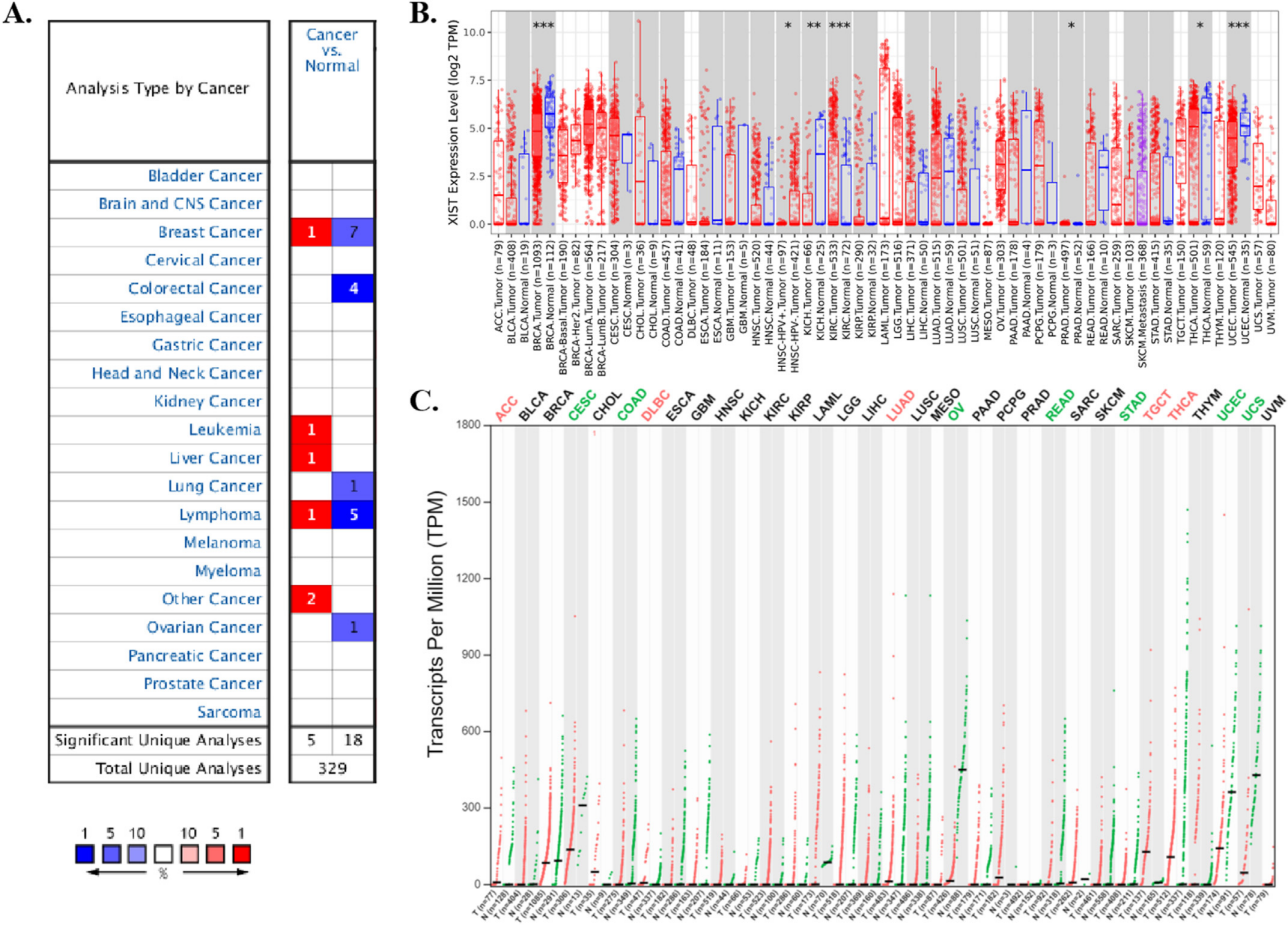
XIST transcript in pan-cancer. **(A)** The transcription levels of XIST in different cancers in Oncomine with exact thresholds (gene rank = 10%, fold change = 2, and *P* < 0.0001). The cell number represented the dataset number with blue for low expression and red for high expression. **(B)** Differential XIST expression between tumors and normal tissues in TIMER 2.0. Red represented tumors and blue represented normal tissues. ∗*P* < 0.05, ∗∗*P* < 0.01, ∗∗∗*P* < 0.001. **(C)** Comparison of XIST expression between tumors and normal tissues in GEPIA2. Compared with normal tissues, red represented cancer samples and significantly higher expression in tumors, green represented normal samples and significantly lower expression in tumors, and black represented no significance.

Three databases, Oncomine, TIMER 2.0 and GEPIA2 displayed the transcription levels of XIST in various types of cancers. In Oncomine, compared to normal tissues, XIST expression was obviously lower in several cancers, such as BRCA, COAD, LUAD, lymphoma and OV (Figure 1A). The details of XIST expression in cancers were shown in Table 1. In TIMER 2.0, XIST expression was also decreased in BRCA in comparison of normal tissues (Figure 1B). In addition, XIST was down-regulated in KICH, THCA and UCEC (Figure 1B). However, it seemed that XIST expression was higher in KIRC and PRAD (Figure 1B). As shown in Figure 1C, the expression levels of XIST seemed to be skimble-scamble in different types of human cancers in GEPIA2. Concretely, XIST expression was decreased significantly in CESC, COAD, OV, READ, STAD, UCEC and UCS; but it was up-regulated in five cancers, including ACC, DLBC, LUAD, TGCT and THCA. However, there were no significant differences between other tumors and normal tissues in GEPIA2, including BRCA. On the whole, XIST was abnormally expressed in different cancers, especially in BRCA, OV, COAD and READ.

**Table 1:** Datasets of XIST transcript across cancers in Oncomine.

Cancer Site	Dataset	Types of Cancer vs Normal	N of normal	N of cancer	Fold change	*t*-test	*p*-value
Breast	TCGA	Male Breast Carcinoma	61	3	-927.150	-38.493	3.13E-9
	Richardson et al.	Ductal Breast Carcinoma	7	40	-3.990	-6.837	9.31E-9
	Curtis et al.	Invasive Breast Carcinoma	144	21	-2.083	-7.369	4.69E-8
		Invasive Ductal Breast Carcinoma	144	1556	-2.124	-20.652	3.87E-52
		Invasive Ductal and Invasive Lobular Breast Carcinoma	144	90	-2.100	-12.619	1.77E-26
		Breast Carcinoma	144	14	-2.195	-6.996	1.72E-6
		Medullary Breast Carcinoma	144	32	-2.420	-8.520	1.09E-10
	Finak et al.	Invasive Breast Carcinoma Stroma	6	53	11.738	18.041	1.50E-24
Colon	Skrzypczak et al.	Colon Adenoma	10	5	-4.884	-16.820	2.40E-10
		Colon Carcinoma	10	5	-6.720	-16.677	2.55E-10
		Colon Adenoma Epithelia	10	5	-10.737	-12.261	1.73E-8
		Colon Carcinoma Epithelia	10	5	-18.031	-14.500	2.58E-9
Blood	Choi et al.∗	Chronic Adult T-Cell Leukemia/Lymphoma	6	19	6.115	5.988	3.90E-6
Liver	Chen et al.	Hepatocellular Adenoma	74	2	12.984	14.540	1.43E-12
Lung	Garber et al.	Lung Adenocarcinoma	6	38	-3.124	-4.837	1.42E-5
Lymph	Eckerle et al.	Primary Cutaneous Anaplastic Large Cell Lymphoma	41	7	-7.205	-7.132	2.93E-9
		Anaplastic Large Cell Lymphoma, ALK-Negative	41	4	-2.951	-5.351	1.60E-6
		Classical Hodgkin's Lymphoma	41	4	-2.251	-5.701	7.60E-7
		Anaplastic Large Cell Lymphoma, ALK-Positive	41	5	-2.914	-4.968	6.71E-6
	Piccaluga et al.	Angioimmunoblastic T-Cell Lymphoma	20	6	-2.333	-4.967	4.94E-5
	Choi et al.∗	Chronic Adult T-Cell Leukemia/Lymphoma	6	19	6.115	5.988	3.90E-6
Ovary	Welsh et al.	Ovarian Serous Surface Papillary Carcinoma	4	28	-10.653	-5.545	2.78E-6
Testis	Sperger et al.	Testicular Seminoma	19	23	14.082	10.531	1.53E-11
	Korkola et al.	Seminoma, NOS	6	12	12.122	8.343	9.28E-7

∗ Choi et al. analyzed Chronic Adult T-Cell Leukemia/Lymphoma and Chronic Adult T-Cell Leukemia/Lymphoma at the same time.

### 3.2 Prognostic value of XIST in human pan-cancer

**Figure 2:**
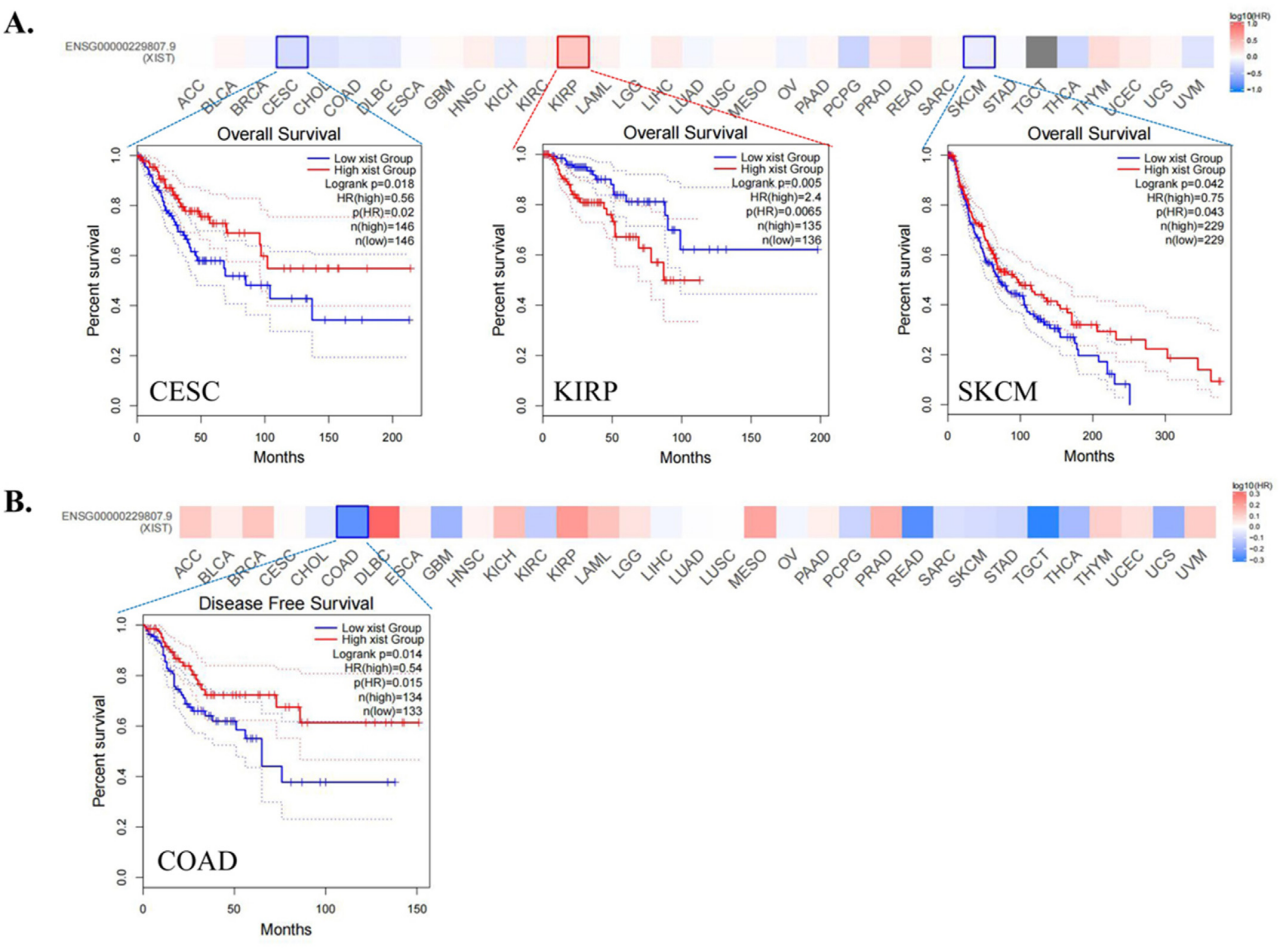
Prognosis analyses of XIST across cancers in GEPIA. **(A)** OS; **(B)** DFS. Red represented high risk and blue represented low risk.

**Figure 3:**
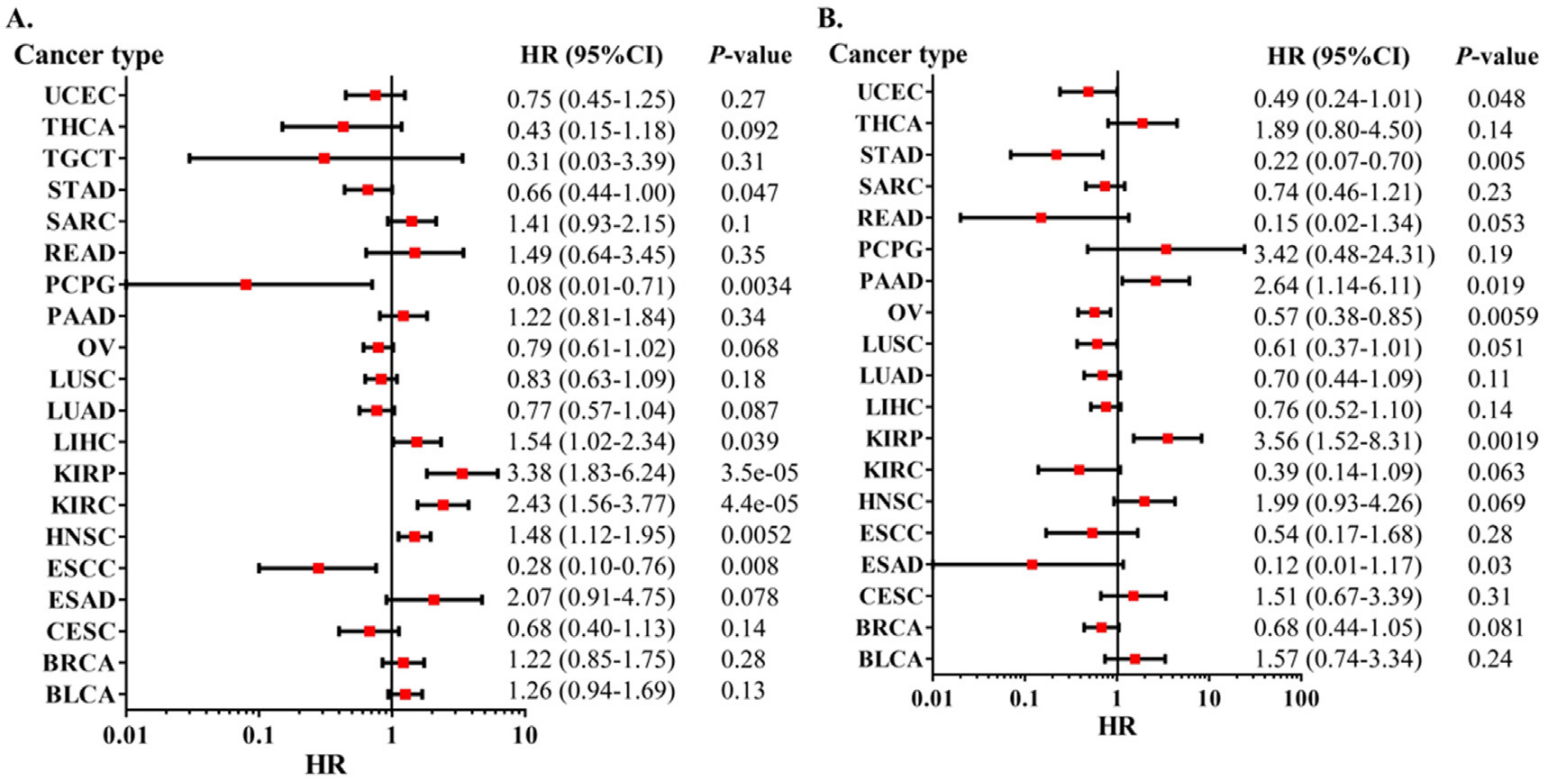
Prognosis analyses of XIST across cancers in Kaplan-Meier Plotter. **(A)** OS; **(B)** RFS.

**Figure 4:**
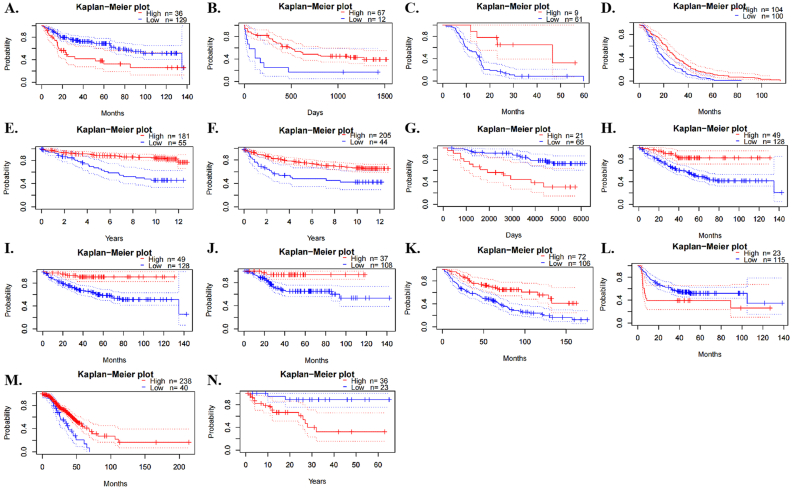
Prognosis analyses of XIST across cancers in PrognoScan. **(A)** OS of BLCA; **(B)** OS of AML; **(C)** OS of GBM; **(D)** RFS of BRCA; **(E)** DSS of BRCA; **(F)** DFS of BRCA; **(G)** DMFS of BRCA; **(H)** OS of COAD; **(I)** DSS of COAD; **(J)** DFS of COAD; **(K)** OS of LUAD; **(L)** RFS of NSCLC; **(M)** OS of OV; **(N)** OS of Renal cell carcinoma.

Then, we analyzed the prognostic role of XIST across cancers in GEPIA2, Kaplan-Meier Plotter and PrognoScan. In GEPIA2, higher expression of XIST indicated longer overall survival rates of patients with CESC and SKCM, but predicted worse prognosis of KIRP (Figure 2A). In addition, XIST expression was positively related to DFS in COAD (Figure 2B). In Kaplan-Meier Plotter, XIST indicated good prognosis of OS in ESCC (*P* = 0.008), PCPG (*P* = 0.0034) and STAD (*P* = 0.047, Figure 3A), and was a favourable factor for RFS in OV (*P* = 0.0059), STAD (*P* = 0.005) and UCEC (*P* = 0.048, Figure 3B). However, XIST was linked to poor outcomes for cancers of HNSC, KIRC, KIRP, LIHC and PAAD. PrognoScan also showed that XIST played an unfavourable prognostic role in several cancers, including BLCA (Figure 4A, OS: Cox *P* = 0.0192), non-small-cell lung cancer (NSCLC, Figure 4L, RFS: Cox *P* = 0.0084) and renal cell carcinoma (RCC, Figure 4N, OS: Cox *P* = 0.0327). But XIST played a protective role in other 6 cancer types, including LAML (Figure 4B, OS: Cox *P* = 0.0324), GBM (Figure 4C, OS: Cox *P* = 0.0285), BRCA (Figure 4D, RFS: Cox *P* = 0.0180; Figure 4E, DSS: Cox *P* = 0.0001; Figure 4F, DFS: Cox *P* = 0.0056), COAD (Figure 4H, OS: Cox *P* = 0.0229; Figure 4I, DSS: Cox *P* = 0.0228; Figure 4J, DFS: Cox *P* = 0.0343), LUAD (Figure 4K, OS: Cox *P* = 0.0004) and OV (Figure 4M, OS: Cox *P* = 0.0074). However, it seemed that XIST was not a favourable factor for DMFS in BRCA (Figure 4G, Cox *P* = 0.0231). The details of survival analyses in PrognoScan were listed in Table 2.

**Table 2:** Prognosis analyses of XIST expression across cancers by univariate Cox regression model in PrognoScan.

CANCER TYPE	DATASET	SUBTYPE	ENDPOINT	N	CUTPOINT	HR [95% CI]	p-VALUE
Bladder cancer	GSE5287		Overall Survival	30	0.57	1.09 [0.88–1.37]	0.424157
	GSE13507		Overall Survival	165	0.78	1.18 [1.03–1.36]	0.019154
		Transitional cell carcinoma	Disease Specific Survival	165	0.82	1.20 [0.99–1.45]	0.060675
Blood cancer	GSE12417-GPL96	AML	Overall Survival	163	0.49	0.98 [0.88–1.09]	0.726558
	GSE12417-GPL97	AML	Overall Survival	163	0.12	1.60 [0.58–4.43]	0.36404
	GSE12417-GPL570	AML	Overall Survival	79	0.15	0.61 [0.39–0.96]	0.032417
	GSE5122	AML	Overall Survival	58	0.19	1.02 [0.87–1.20]	0.776297
	GSE8970	AML	Overall Survival	34	0.24	0.86 [0.68–1.09]	0.21609
	GSE4475	B-cell lymphoma	Overall Survival	158	0.23	1.03 [0.86–1.22]	0.769231
	E-TABM-346	DLBCL	Overall Survival	53	0.85	1.11 [0.91–1.36]	0.295587
		DLBCL	Event Free Survival	53	0.85	1.20 [0.99–1.45]	0.064109
	GSE16131-GPL96	Follicular lymphoma	Overall Survival	180	0.21	1.02 [0.96–1.09]	0.440844
	GSE16131-GPL97	Follicular lymphoma	Overall Survival	180	0.86	1.01 [0.95–1.08]	0.769404
	GSE2658	Multiple myeloma	Disease Specific Survival	559	0.19	0.98 [0.88–1.10]	0.751994
Brain cancer	GSE4271-GPL96	Astrocytoma	Overall Survival	77	0.4	0.94 [0.82–1.08]	0.395516
	GSE4271-GPL97	Astrocytoma	Overall Survival	77	0.27	0.87 [0.68–1.11]	0.272633
	GSE7696	Glioblastoma	Overall Survival	70	0.87	0.33 [0.13–0.89]	0.028511
	MGH-glioma	Glioma	Overall Survival	50	0.44	1.00 [0.73–1.37]	0.984826
	GSE4412-GPL96	Glioma	Overall Survival	74	0.73	0.88 [0.76–1.02]	0.094787
	GSE4412-GPL97	Glioma	Overall Survival	74	0.62	0.91 [0.83–1.01]	0.073693
	GSE16581	Meningioma	Overall Survival	67	0.84	68.57 [2.29–2053.81]	0.014788
Breast cancer	GSE19615		Distant Metastasis Free Survival	115	0.53	0.79 [0.45–1.38]	0.407813
	GSE3143		Overall Survival	158	0.72	1.02 [0.68–1.53]	0.931487
	GSE7849		Disease Free Survival	76	0.54	1.29 [0.57–2.94]	0.536705
	GSE12276		Relapse Free Survival	204	0.49	0.77 [0.62–0.96]	0.018036
	GSE6532-GPL570		Relapse Free Survival	87	0.76	5.11 [1.25–20.89]	0.02312
			Distant Metastasis Free Survival	87	0.76	5.11 [1.25–20.89]	0.02312
	GSE9195		Distant Metastasis Free Survival	77	0.52	0.85 [0.12–6.07]	0.868331
			Relapse Free Survival	77	0.82	0.84 [0.50–1.42]	0.519039
	GSE12093		Distant Metastasis Free Survival	136	0.34	0.88 [0.50–1.57]	0.670572
	GSE11121		Distant Metastasis Free Survival	200	0.24	0.79 [0.54–1.16]	0.2262
	GSE1378		Relapse Free Survival	60	0.6	1.03 [0.82–1.30]	0.796121
	GSE1379		Relapse Free Survival	60	0.45	0.88 [0.59–1.30]	0.514418
	GSE2034		Distant Metastasis Free Survival	286	0.77	0.98 [0.74–1.30]	0.905737
	GSE1456-GPL96		Overall Survival	159	0.4	0.82 [0.53–1.28]	0.391112
			Relapse Free Survival	159	0.48	0.64 [0.43–0.94]	0.023968
			Disease Specific Survival	159	0.4	0.64 [0.40–1.03]	0.063692
	GSE1456-GPL97		Overall Survival	159	0.53	0.83 [0.56–1.22]	0.334743
			Relapse Free Survival	159	0.53	0.62 [0.45–0.86]	0.004468
			Disease Specific Survival	159	0.53	0.62 [0.42–0.91]	0.013672
	GSE7378		Disease Free Survival	54	0.56	0.97 [0.63–1.50]	0.900162
	E-TABM-158		Overall Survival	117	0.74	1.07 [0.73–1.59]	0.7209
			Distant Metastasis Free Survival	117	0.74	1.35 [0.79–2.33]	0.272623
			Relapse Free Survival	117	0.74	1.07 [0.73–1.59]	0.7209
			Disease Specific Survival	117	0.74	1.22 [0.76–1.96]	0.418075
	GSE3494-GPL96		Disease Specific Survival	236	0.17	0.43 [0.28–0.64]	0.000054
	GSE3494-GPL97		Disease Specific Survival	236	0.23	0.34 [0.21–0.55]	0.000009
	GSE4922-GPL96		Disease Free Survival	249	0.17	0.68 [0.46–0.99]	0.043122
	GSE4922-GPL97		Disease Free Survival	249	0.1	0.53 [0.35–0.81]	0.003717
	GSE2990		Relapse Free Survival	62	0.16	0.81 [0.61–1.07]	0.144443
			Distant Metastasis Free Survival	125	0.49	1.00 [0.64–1.56]	0.985741
	GSE7390		Overall Survival	198	0.15	1.00 [0.84–1.18]	0.974118
			Relapse Free Survival	198	0.15	0.96 [0.84–1.10]	0.555227
			Distant Metastasis Free Survival	198	0.15	0.99 [0.84–1.16]	0.901166
Colorectal cancer	GSE12945		Disease Free Survival	51	0.33	1.02 [0.70–1.50]	0.899923
			Overall Survival	62	0.26	1.10 [0.87–1.40]	0.424702
	GSE17536		Disease Specific Survival	177	0.72	0.29 [0.10–0.84]	0.022846
			Overall Survival	177	0.54	0.31 [0.11–0.86]	0.024521
			Disease Free Survival	145	0.74	0.25 [0.07–0.90]	0.034334
	GSE14333		Disease Free Survival	226	0.11	0.97 [0.88–1.06]	0.485628
	GSE17537		Disease Specific Survival	49	0.27	0.02 [0.00–0.74]	0.033358
			Overall Survival	55	0.11	0.07 [0.01–0.73]	0.026241
			Disease Free Survival	55	0.31	0.09 [0.01–1.03]	0.053347
Esophagus cancer	GSE11595	Adenocarcinoma	Overall Survival	34	0.38	0.24 [0.05–1.12]	0.070138
Eye cancer	GSE22138	Uveal melanoma	Distant Metastasis Free Survival	63	0.14	0.92 [0.79–1.07]	0.294872
Head and neck cancer	GSE2837	Squamous cell carcinoma	Relapse Free Survival	28	0.68	0.45 [0.01–36.57]	0.722659
Lung cancer	jacob-00182-CANDF	Adenocarcinoma	Overall Survival	82	0.15	0.85 [0.66–1.09]	0.192625
	jacob-00182-HLM	Adenocarcinoma	Overall Survival	79	0.9	1.01 [0.89–1.15]	0.835337
	jacob-00182-MSK	Adenocarcinoma	Overall Survival	104	0.44	0.99 [0.83–1.18]	0.866824
	GSE13213	Adenocarcinoma	Overall Survival	117	0.62	0.95 [0.91–1.00]	0.053709
	GSE31210	Adenocarcinoma	Overall Survival	204	0.5	0.93 [0.81–1.07]	0.290452
		Adenocarcinoma	Relapse Free Survival	204	0.1	0.95 [0.89–1.01]	0.131322
	jacob-00182-UM	Adenocarcinoma	Overall Survival	178	0.6	0.81 [0.73–0.91]	0.000391
	GSE3141	NSCLC	Overall Survival	111	0.83	1.06 [0.91–1.25]	0.439928
	GSE14814	NSCLC	Overall Survival	90	0.57	0.96 [0.69–1.32]	0.798698
		NSCLC	Disease Specific Survival	90	0.12	1.05 [0.75–1.46]	0.795286
	GSE4716-GPL3694	NSCLC	Overall Survival	50	0.2	0.74 [0.41–1.33]	0.312198
	GSE4716-GPL3696	NSCLC	Overall Survival	50	0.84	0.56 [0.28–1.11]	0.096181
	GSE8894	NSCLC	Relapse Free Survival	138	0.83	2.57 [1.27–5.19]	0.008419
	GSE4573	Squamous cell carcinoma	Overall Survival	129	0.87	0.90 [0.75–1.07]	0.223413
Ovarian cancer	GSE9891		Overall Survival	278	0.14	0.51 [0.32–0.84]	0.007439
	DUKE-OC		Overall Survival	133	0.14	0.87 [0.79–0.96]	0.00412
	GSE26712		Disease Free Survival	185	0.78	0.92 [0.81–1.03]	0.152434
			Overall Survival	185	0.75	0.92 [0.81–1.05]	0.232231
	GSE17260		Progression Free Survival	110	0.15	0.93 [0.82–1.06]	0.29524
			Overall Survival	110	0.15	0.91 [0.77–1.07]	0.245215
	GSE14764		Overall Survival	80	0.34	0.74 [0.54–1.01]	0.058791
Renal cell carcinoma	E-DKFZ-1		Overall Survival	59	0.39	2.37 [1.07–5.21]	0.032722
Skin cancer	GSE19234	Melanoma	Overall Survival	38	0.13	1.07 [0.82–1.41]	0.61023
Soft tissue cancer	GSE30929	Liposarcoma	Distant Recurrence Free Survival	140	0.63	0.99 [0.88–1.11]	0.862006

### 3.3 Interactions of XIST with proteins and miRNAs across cancers

**Figure 5:**
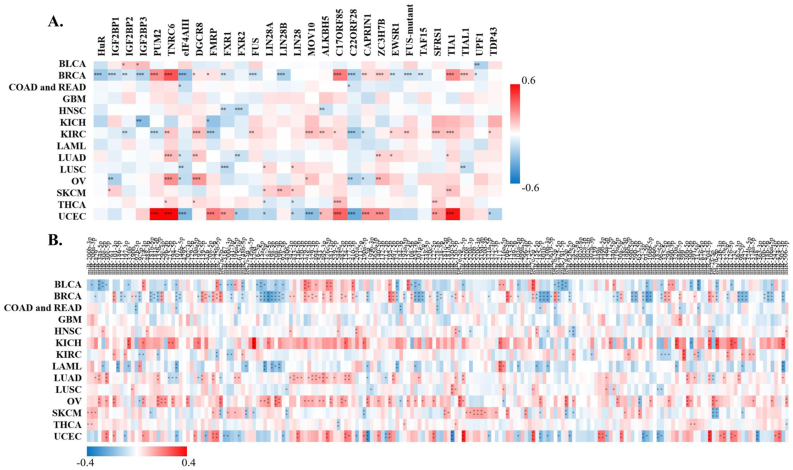
Correlations of XIST with predicted XIST-interacting proteins and miRNAs in starBase. **(A)** Predicted XIST-interacting proteins; **(B)** Predicted XIST-interacting miRNAs. Red represented positive correlation and blue represented negative correlation. ∗*P* < 0.05, ∗∗*P* < 0.01, ∗∗∗*P* < 0.001.

**Figure 6:**
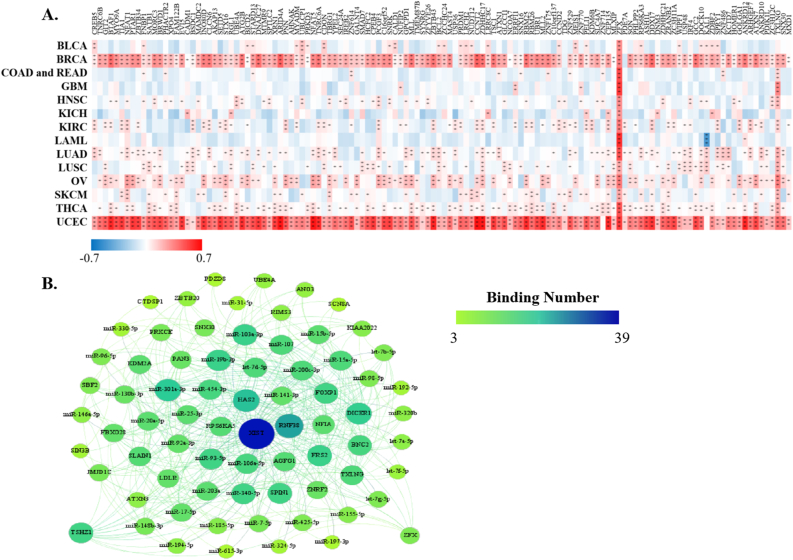
Correlations of XIST with ceRNAs in starBase. **(A)** ceRNAs. Red represented positive correlation and blue represented negative correlation. ∗*P* < 0.05, ∗∗*P* < 0.01, ∗∗∗*P* < 0.001. **(B)** A ceRNA network.

StarBase discovered that XIST could potentially interact with 29 proteins (Figure 5A) and 191 miRNAs (Figure 5B) across cancers. Over 8 proteins were significantly related to XIST in 4 types of cancers, including BRCA, KIRC, OV and UCEC (Figure 5A). Among them, TNRC6, DGCR8, C17ORF85, ZC3H7B, SFRS1 and TIA1, were obviously positively associated with XIST in ≥3 cancers; while eIF4AIII, FXR1, FXR2 and C22ORF28 were significantly negatively correlated with XIST in ≥3 cancers (Figure 5A). Although these miRNAs might be sponged by XIST according to the prediction of LncRNA - miRNA interactions from starBase, their expression levels were different among tumors (Figure 5B). Over 10 miRNAs were negatively related to XIST in KIRC, LAML, COAD and READ, while over 10 miRNAs were positively associated with XIST in KICH, LUAD and OV (Figure 5B). In addition, there were more than a dozen miRNAs both negatively and positively correlated with XIST (≥10, respectively) in BLCA, BRCA, SKCM and UCEC (Figure 5B). Then, we selected out 4 miRNAs, including miR-103a-3p, miR-107, miR-130b-3p and miR-96–5p, which were all negatively related to XIST expression in more than 3 types of cancers. Furthermore, 128 ceRNAs were found to compete with XIST for miRNAs binding and were positively related to XIST across cancers, especially BRCA and UCEC (Figure 6A). Particularly, expressions of ZFX and TXLNG were both positively correlated with XIST in over 10 cancers. According to TargetScan, miRBase and starBase, we scheduled a network of LncRNA - miRNAs - mRNAs (Figure 6B).

### 3.4 Correlation between XIST and immune infiltration levels in multiple cancers

**Figure 7:**
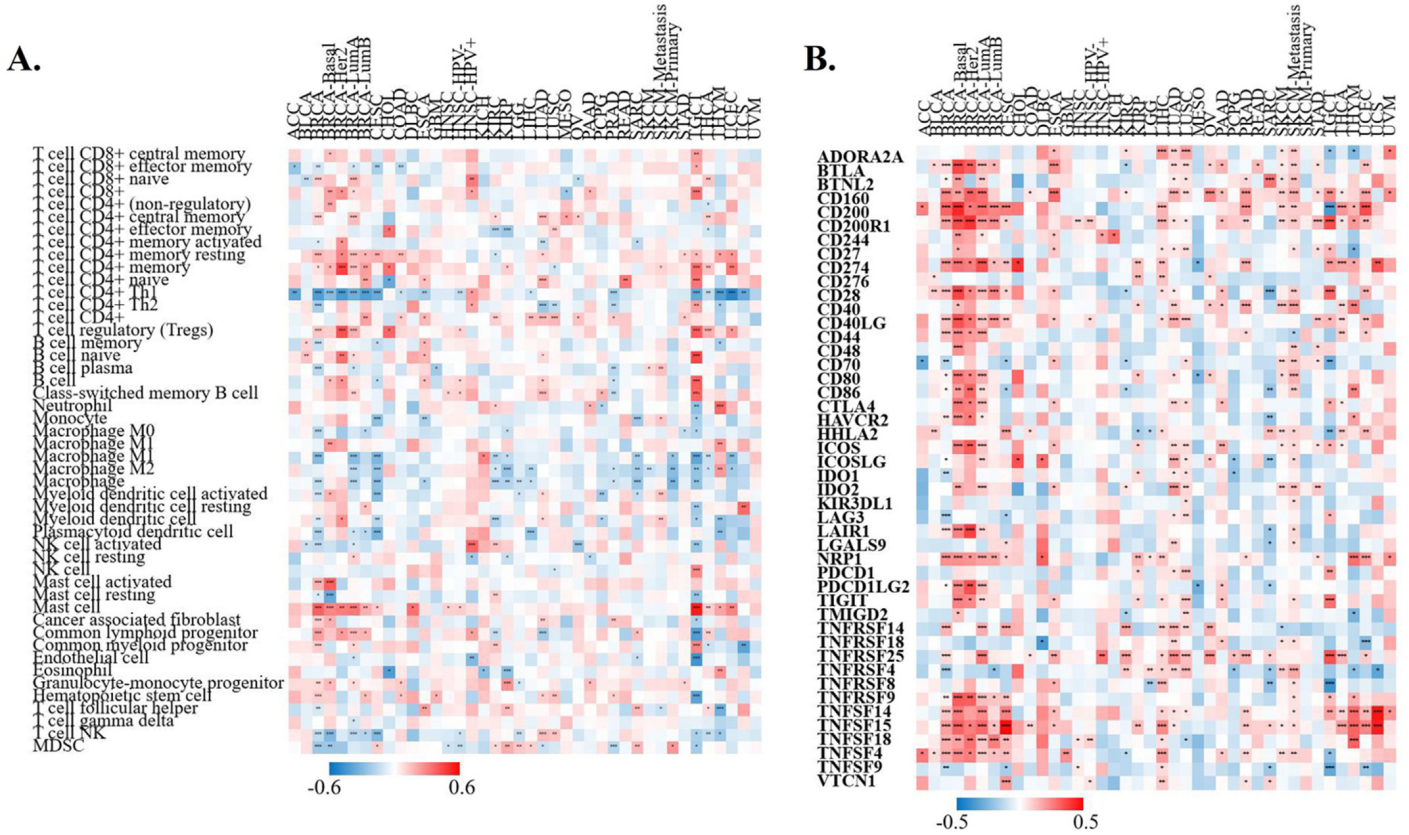
Correlations of XIST with immune infiltration levels and checkpoint markers across cancers in TIMER2. **(A)** Immune infiltration levels; **(B)** Immune checkpoint markers. Red represented positive correlation and blue represented negative correlation. ∗*P* < 0.05, ∗∗*P* < 0.01, ∗∗∗*P* < 0.001.

To determine the role of XIST in TIME, we analyzed the correlation of XIST expression with immune infiltration through TIMER 2.0 database. Its expression was positively related to infiltration levels of T cell CD4+ memory resting, T cell CD4+ memory, T cell CD4+, Tregs and mast cell in over 8 types of cancers, while it was negatively correlated with T cell CD4+ Th1, Macrophage and T cell NK in more than 8 types (Figure 7A). Hence, XIST might influence immune infiltration in the TME.

### 3.5 Correlation between XIST and immune checkpoint markers in multiple cancers

**Figure 8:**
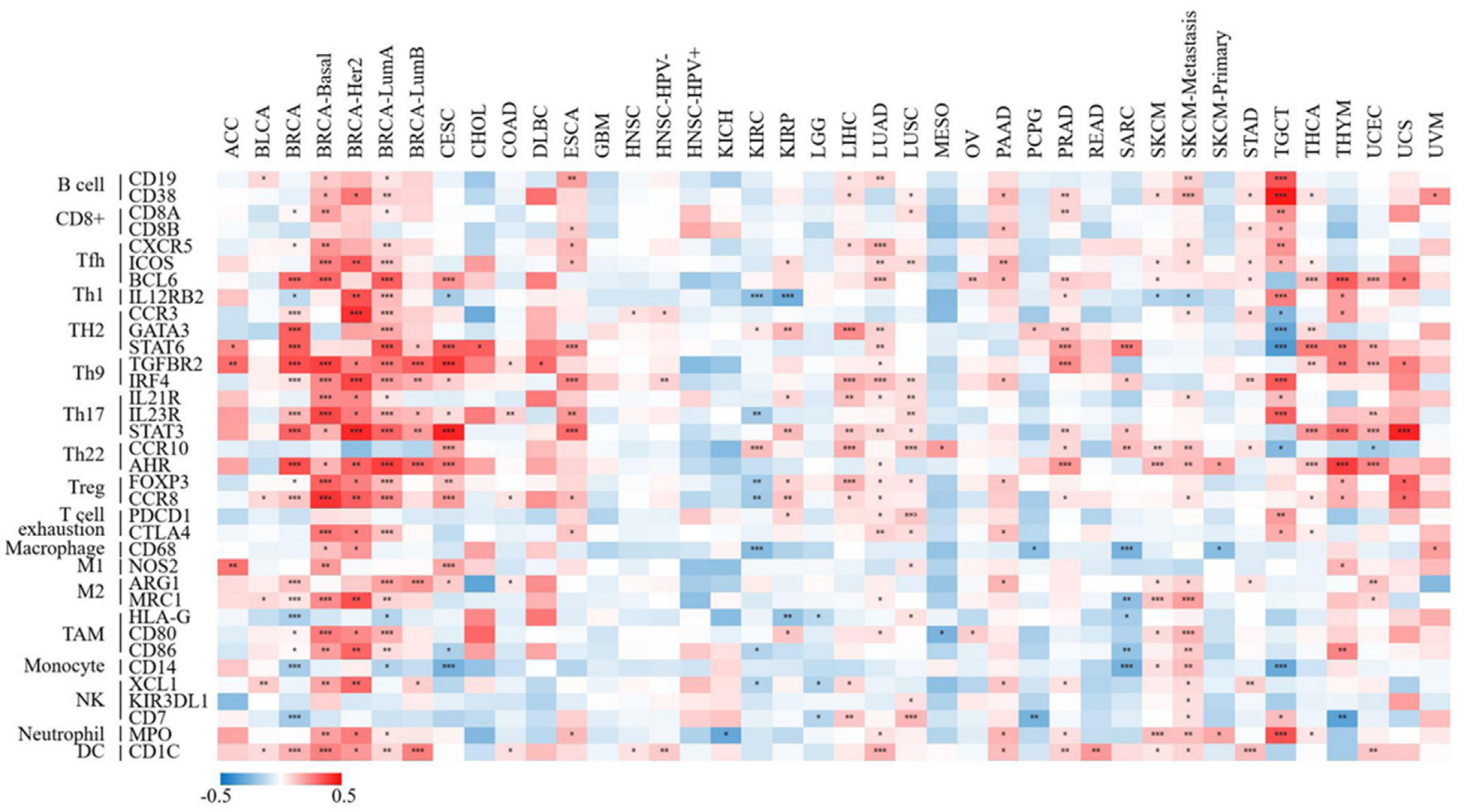
Correlations of XIST with immune cell types in multiple cancers in TIMER2. Red represented positive correlation and blue represented negative correlation. ∗*P* < 0.05, ∗∗*P* < 0.01, ∗∗∗*P* < 0.001.

To investigate the immunoregulatory mechanism of XIST, we investigated more than 40 common immune checkpoint markers in TIMER 2.0 across cancers. Generally, XIST expression was positively related to about half of these markers in various cancers, such as CD2000, CD200R1, CD274, members of the tumor necrosis factor (TNF) ligand family, and so on (Figure 7B). Figure 8 showed the correlation of XIST expression with 35 immune cell types. Notably, its expression was positively related to most of these cell types (>20) in BRCA. In addition, XIST expression was also positively associated with over 10 cell types in CESC, ESCA, LIHC, LUAD, LUSC, PAAD, PRAD, SKCM-Metastasis, STAD, TGCT and THYM. However, XIST expression was negatively linked with some immune cell types in several cancers, including KIRC (7 cell types), SARC (5 cell types) and TGCT (5 cell types). These results implied that abnormal expression of XIST might become a vital factor for survival of cancer patients through its regulation on the TIME.

### 3.6 Correlation between XIST and representative gene mutation across cancers

**Figure 9:**
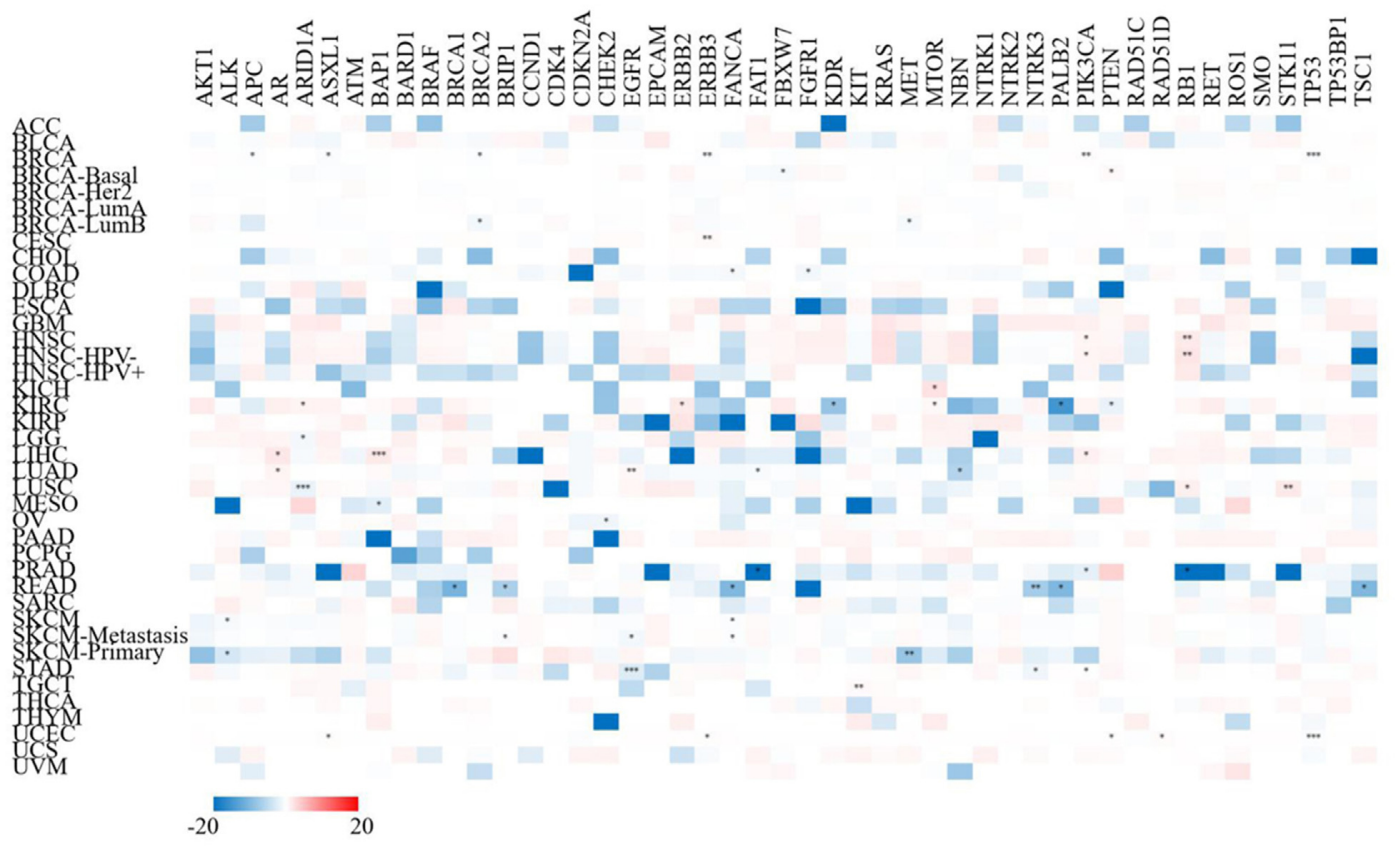
Correlations of XIST with mutations of representative tumor-associated genes across cancers in TIMER2. Red represented positive correlation and blue represented negative correlation. ∗*P* < 0.05, ∗∗*P* < 0.01, ∗∗∗*P* < 0.001.

Next, we analyzed the relationship of XIST expression with 47 types of tumor-associated gene mutation to further explore the mechanism of carcinogenesis of dysregulation of XIST. Generally, theses mutations were weakly related to only several types of cancers (<5 cancer types for each gene mutation, Figure 9). However, in PRAD, XIST expression was significantly negatively associated with mutations of FAT1 and RB1. In READ, its expression was negatively related to 6 gene mutations, including BRCA1, BRIP1, FANCA, NTRK3, PALB2 and TSC1. In addition, XIST was slightly negatively associated with 5 gene mutations in BRCA (APC, ASXL1, BRCA2, ERBB3 and TP53), but positively related to mutation of PIK3CA. These findings reflected that XIST expression might be affected by tumor-associated gene mutation in several cancers, especially BRCA and READ.

### 3.7 Roles of XIST promoter methylation in expression and prognosis

**Figure 10:**
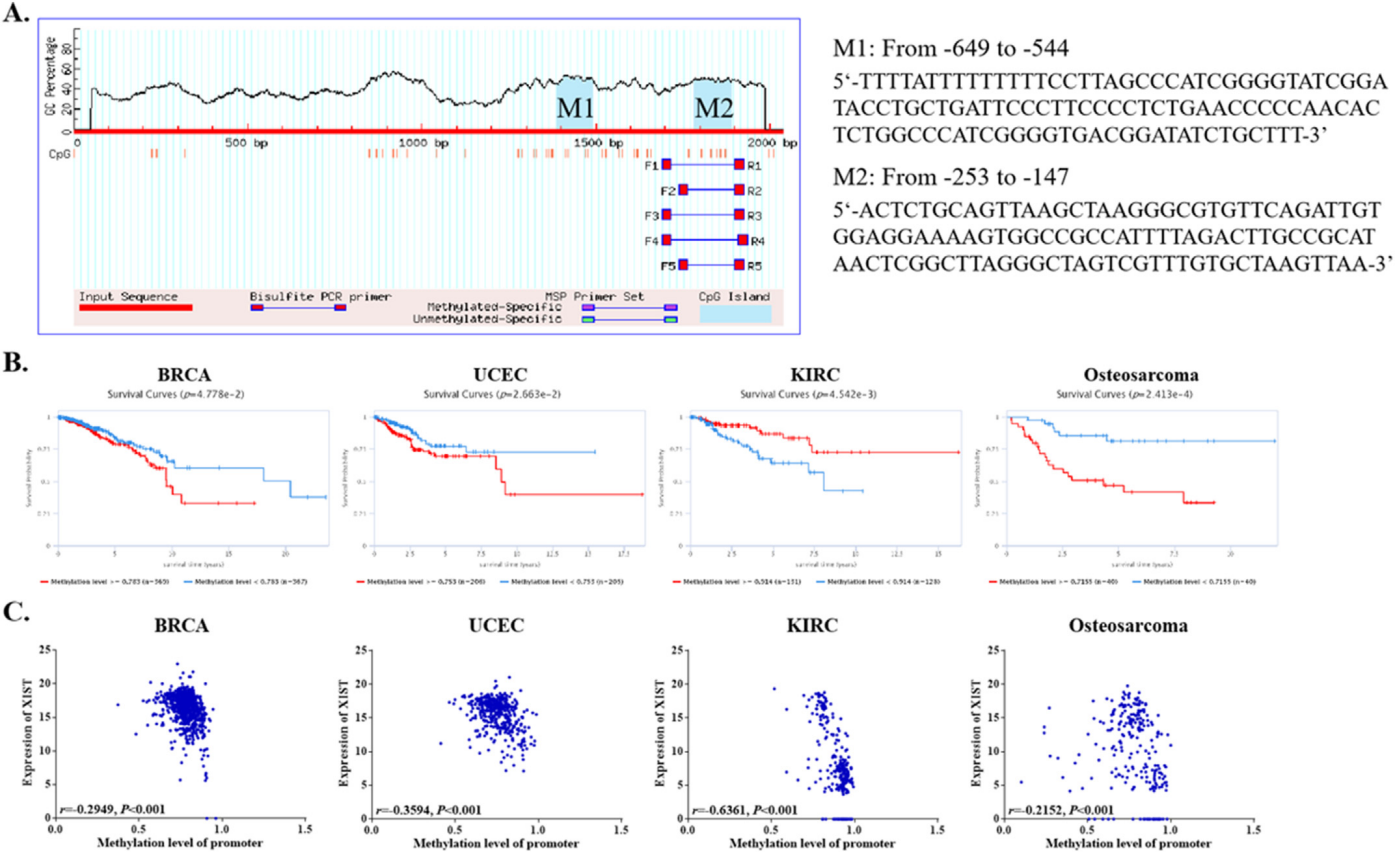
Methylation analysis of XIST promoter. **(A)** MethPrimer analysis of XIST promoter; **(B)** Significant differences of survival time between high and low XIST promoter methylation in EWAS Data Hub; **(C)** Correlation between XIST promoter methylation and its expression in EWAS Data Hub.

From EPD and MethPrimer, we identified two CpG islands in XIST promoter (Figure 10A). Among 39 types of cancers, only four cancers, BRCA, UCEC, KIRC and osteosarcoma, had differences of survival time between high and low methylation levels (Figure 10B). Concretely, higher methylation level of XIST indicated shorter overall survival rates of patients with BRCA, UCEC and osteosarcoma, but predicted better prognosis of KIRC (Figure 10B). In these four cancers, XIST promoter methylation was negatively correlated with XIST expression, which indicated that methylation of XIST promoter might contribute to down-regulated expression of XIST in cancers (Figure 10C).

### 3.8 Clinicopathological role of XIST in breast cancer

**Figure 11:**
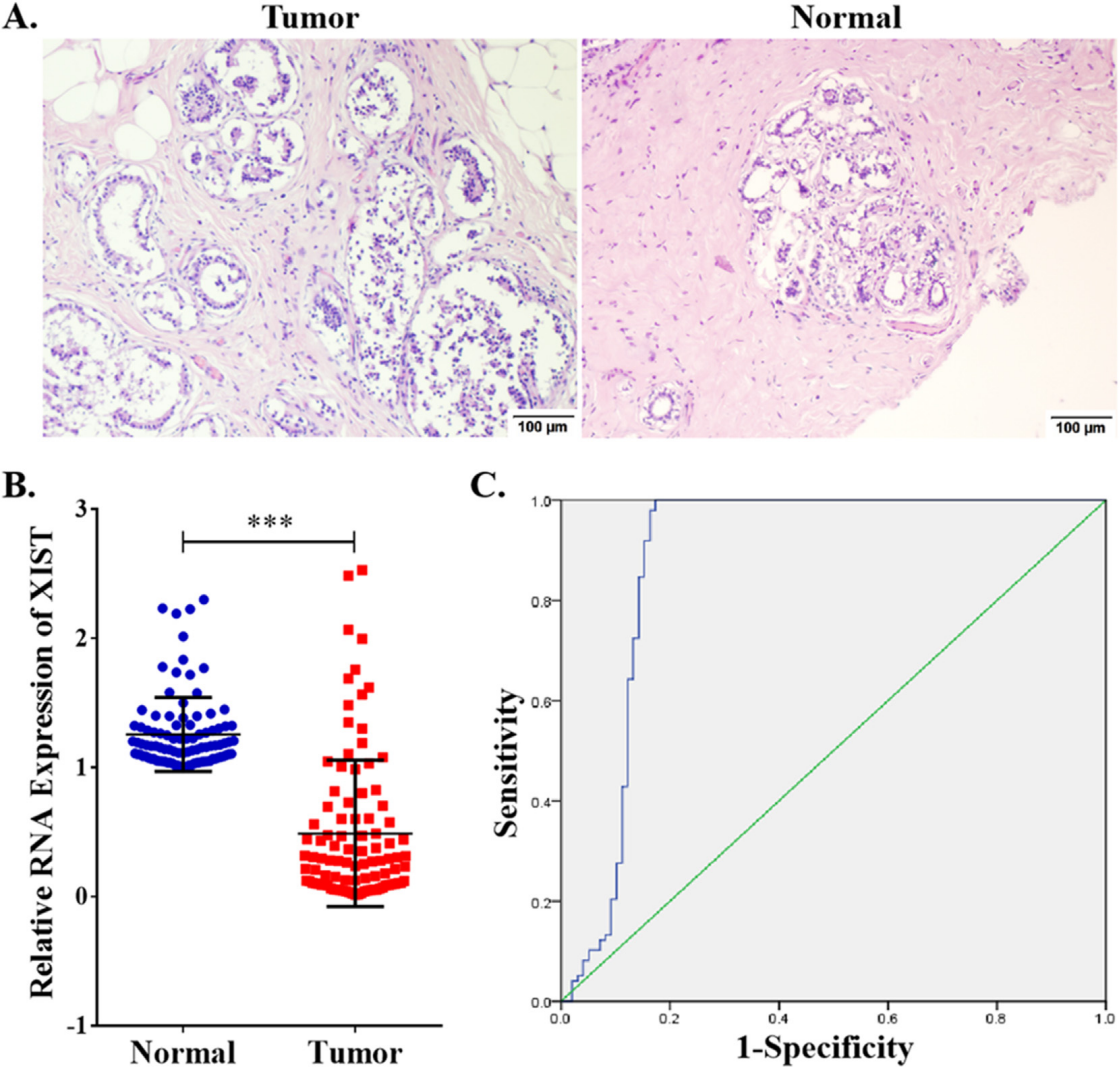
Correlation analysis of XIST between breast tumors and normal tissues. **(A)** Microscopic observation of breast cancer and adjacent tissues with HE staining (×100); **(B)** Comparison of XIST expression between breast cancer and normal tissues; **(C)** The ROC curve of XIST. ∗∗∗*P* < 0.001.

Due to these functions of XIST in BRCA in public databases, we selected 98 breast cancer samples to assess its clinicopathological role. Tumors and normal tissues were observed by HE staining (Figure 11A). Compared to normal tissues, XIST expression was obviously lower via qRT-PCR (*t* = 13.05, *P* < 0.001, Figure 11B). According to its expression, a ROC curve was conducted in Figure 11C. The AUC (Area Under The Curve) value of XIST was 0.893 (95%CI: 0.837–0.950). The maximum value of Youden's index was 0.827 and the corresponding XIST expression was 0.9928. Thus, samples with expression value ≥0.9928 were included into “positive group” and those with expression value <0.9928 were included into “negative group”. As shown in Table 3, expression of XIST was negatively related to lymph node invasion (*P* = 0.004) and TNM stage (*P* = 0.029), but was unrelated to age, tumor size and histopathologic grade.

**Table 3:** Relationship between XIST expression and clinicopathological parameters of breast cancer.

Parameters	n	XIST positive expression	χ^2^	*p*
Total	98	17		
Year				
≤55	58	9	0.332	0.565
>55	40	8		
Tumor size				
≤2cm	32	8	2.728	0.256
2–5 cm	34	6		
＞5 cm	32	3		
Histological grade				
1	26	5	4.050	0.132
2	41	10		
3	31	2		
Lymph node invasion				
No	29	10	8.435	0.004
Yes	69	7		
TNM stage				
I	12	4	9.059	0.029
II	50	12		
III	29	1		
IV	7	0		

## 4 Discussion

LncRNAs were initially described as mere “transcriptional noise”, but now increasing studies have demonstrated that LncRNAs act as critical modulators in a variety of physiological activities [37]. However, only a relatively limited number of LncRNAs have been confirmed to have critical biological functions, and molecular mechanisms of most LncRNAs have not been illustrated [38]. XIST is a pivotal initiator of imprinted and random X-chromosome inactivation in mammals, and it silences one X chromosome in order to avoid the excessive activation of genes [39]. When dysregulation of XIST occurs, varieties of diseases will emerge due to the escape from X chromosome inactivation [39]. But its mechanisms for pathogenesis are more than that. Recent studies have demonstrated that loss of XIST promotes tumor growth and invasion of BRCA due to the reduction of endogenous competition against onco-miRNAs [16, 40]. However, its functions on tumors were not consistent in previous studies. Several researches showed that growth and metastasis of cancer cells were facilitated due to the abnormal upregulation of XIST [17, 18, 41]. This inconformity resulted in failure to determine whether XIST could become a strong prognostic indicator of cancer patients.

From three databases, Oncomine, TIMER and GEPIA, we found relative expression levels of XIST were different among multiple cancers. Particularly, XIST was down-regulated and predicted a good prognosis in BRCA, COAD and OV, but its expression was inconsistent with outcomes in other cancers. For instance, prognostic roles of XIST between BRCA and kidney neoplasms were opposite, which might attribute to different types of cells, sources of germ layers and effects of hormone. Therefore, we demonstrated that XIST might become a good molecular biological indicator for prognosis of patients with BRCA, COAD and OV, though with the heterogeneity of prognostic results among different databases. Our research also showed that XIST expression was obviously lower than that in normal tissues from 98 breast cancer samples and its expression was negatively related to lymph node invasion and TNM stage. However, several studies showed that XIST was up-regulated in these tumors and was negatively linked with survival rates of patients [42, 43, 44]. Hence, more samples are essential to further researches which will be conducted to verify the prognostic roles of XIST in other cancers.

It is well known that LncRNAs can function as sponges for multiple tumor-associated miRNAs and indirectly regulate expression of targeted mRNAs [13]. In this research, 191 miRNAs were found to be possibly interacted with XIST. However, not all of them were negatively related to XIST expression, which might be affected by other factors. Only 4 miRNAs, including miR-103a-3p, miR-107, miR-130b-3p and miR-96–5p, were negatively linked with XIST in more than 3 types of cancers. All of these 4 miRNAs played promoting roles in carcinogenesis by targeting mRNAs [45, 46, 47, 48]. Therefore, XIST might function as a tumor suppressor LncRNA by sponging these onco-miRNAs. In addition, these miRNAs could be in turn interacting with lncRNA XIST, which suggested that up-regulation of them might repress XIST expression in tumor cells.

LncRNAs also played fundamental roles in diverse biological and pathological processes by interacting with specific proteins [3]. To search the proteins combined with XIST, we performed the starBase database and preliminarily determined 29 candidate proteins. Among them, only 6 ones were co-expressed with XIST, including TNRC6, DGCR8, C17ORF85 (NCBP3), ZC3H7B, SFRS1 and TIA1, all of which were involved in the development and progression of diverse tumors [49, 50, 51, 52, 53, 54]. Thus, we identified that these six proteins potentially interacted with XIST. However, further studies need to be performed to confirm whether these proteins can cooperate with XIST to affect biological behaviors and epigenetic pathways of cancers.

Currently, XIST is emerging as a critical immune-related LncRNA and silences a subset of X-linked immune genes [55]. The escape of XIST-dependent genes induced the genesis and recurrence of tumors through diverse perplexing mechanisms [55]. This present research found that XIST was related to infiltration levels of T cell CD4+, Tregs, mast cell, Th1, macrophage and T cell NK in more than 8 types of cancers. One previous study of early-stage LUAD showed that XIST was positively linked with B cells, dendritic cells, follicular helper T cells, mast cells, T cell CD4+/CD8+ effector memory and eosinophil, but was negatively correlated with macrophage, Th2 [56]. In addition, XIST expression was positively related to more than 20 immune checkpoint markers and over 10 immune cell types across cancers. Therefore, we demonstrated that XIST could become a immune-related LncRNA and played a pivotal role in immune-oncology.

Genetic variation is associated with various diseases, especially carcinomas [26]. Mutations of tumor-associated genes appear in carcinomas, including base substitution and frameshift mutation, and regulate expression of downstream genes [26]. Cancer is essentially a genetic disease where cells either die or cancerate, when a certain number of genetic mutations accumulate in the cells. Our research also showed that XIST might be regulated by gene mutation (including APC, BRCA1, BRCA2, TP53 and PIK3CA) in several cancers, especially BRCA, PRAD and READ. Mutations of these genes modulate expressions of diverse downstream genes and have become important therapeutic targets for anticancer drug development [57, 58]. For instance, PARP inhibitors have been used for cancer patients with BRCA1/BRCA2 mutation. The XIST RNA domain number in BRCA1 breast tumor was associated with chromosomal genetic abnormalities, and XIST might become a predictive biomarker for prognosis of patients with BRCA1 breast tumor [27, 59, 60]. But BRCA1 was dispensable for XIST RNA coating of the X chromosome [59]. These findings provided clues on the association between XIST and mutation of tumor-associated genes. Hence, future researches should be conducted to explore possible mechanisms of XIST in multiple cancers.

Besides gene mutation, promoter methylation also contributes to regulation of genes [61]. Our research revealed that expression of XIST was negatively linked with its promoter methylation level. Notably, in BRCA, high level of XIST promoter methylation correlated with a poor prognosis, while high expression of XIST predicted a well outcome. Thus, in a sense, low expression of XIST might arise from its promoter methylation, which became an important factor for progression and metastasis of tumors, especially BRCA. Therefore, we need to conduct further researches to verify the effect of XIST promoter methylation. In addition, methylation of promoter of XIST theoretically correlated with immune infiltration. However, no databases were found to access relationships between methylation of promoter and immune infiltration in cancers. Therefore, more researches, like chromatin immunoprecipitation, need to be conducted to confirm the correlation between methylation of XIST promoter and immune infiltration in BRCA.

Admittedly, this research still had some limitations. First, it was difficult to analyze the prognostic value of XIST in sub-types of cancers through public datasets. Secondly, since this study was based on pan-cancer data, we failed to prove all the ideas at the same time. Thirdly, our results lacked external validation in other public databases or in vitro and in vivo researches. This theoretical work remains to be verified. For example, more cancer patients will be selected out and divided into groups by sub-types with 5-year or 10-year follow-up, so that we can confirm the prognostic roles both of expression and methylation of XIST in sub-types of cancers.

## 5 Conclusion

In conclusion, dysregulation of LncRNA XIST was significantly associated with prognosis, miRNAs, immune cell infiltration, mutations of tumor-associated genes and its promoter methylation in multiple cancers, especially BRCA and colorectal cancer. XIST may act as a novel biomarker for survival and immunotherapy across cancers in the immediate future.

## Declarations

### Author contribution statement

Wei Han: Conceived and designed the experiments; Performed the experiments; Analyzed and interpreted the data; Contributed reagents, materials, analysis tools or data; Wrote the paper.

Chun-tao Shi; Jun Ma: Performed the experiments; Contributed reagents, materials, analysis tools or data; Wrote the paper.

Hua Chen; Ying Zhou; Jing-feng Gu: Performed the experiments.

Qi-xiang Shao: Performed the experiments; Analyzed and interpreted the data; Contributed reagents, materials, analysis tools or data.

Xiao-jiao Gao: Performed the experiments; Analyzed and interpreted the data.

Hao-nan Wang: Conceived and designed the experiments; Analyzed and interpreted the data.

### Funding statement

Dr. Hao-nan Wang was supported by 10.13039/501100008109Wuxi Municipal Bureau on Science and Technology [Y20212039].

Wei Han was supported by Suzhou Scientific and Technological Development Plan (People's Livelihood Science and Technology - Basic Research Project of Medical Treatment and Health Application) [SYS2020060], Kunshan Major Project of Social Research and Development [KS19038].

Qi-xiang Shao was supported by High-Level Medical Talents in Kunshan [ksgccrc2007].

### Data availability statement

Data included in article/supplementary material/referenced in article.

### Declaration of interest’s statement

The authors declare no conflict of interest.

### Additional information

No additional information is available for this paper.
